# Cancer-associated *SF3B1* mutations affect alternative splicing by promoting alternative branchpoint usage

**DOI:** 10.1038/ncomms10615

**Published:** 2016-02-04

**Authors:** Samar Alsafadi, Alexandre Houy, Aude Battistella, Tatiana Popova, Michel Wassef, Emilie Henry, Franck Tirode, Angelos Constantinou, Sophie Piperno-Neumann, Sergio Roman-Roman, Martin Dutertre, Marc-Henri Stern

**Affiliations:** 1 Department of Genetics and Biology of Cancers, INSERM U830, Institut Curie, PSL Research University, Paris 75248, France; 2 Depatment of Developmental Biology and Genetics, CNRS UMR 3215/INSERM U934, Institut Curie, PSL Research University, Paris 75248, France; 3 Translational Research Department, Institut Curie, PSL Research University, Paris 75248, France; 4 Department of Molecular Bases of Human Diseases, CNRS UPR 1142, IGH-Institute of Human Genetics, Montpellier 34090, France; 5 Department of Medical Oncology, Institut Curie, Paris 75248, France; 6 Department of Genotoxic stress and Cancer, CNRS UMR 3348, Institut Curie, PSL Research University, Orsay 91400, France

## Abstract

Hotspot mutations in the spliceosome gene *SF3B1* are reported in ∼20% of uveal melanomas. SF3B1 is involved in 3′-splice site (3′ss) recognition during RNA splicing; however, the molecular mechanisms of its mutation have remained unclear. Here we show, using RNA-Seq analyses of uveal melanoma, that the *SF3B1*^R625/K666^ mutation results in deregulated splicing at a subset of junctions, mostly by the use of alternative 3′ss. Modelling the differential junctions in *SF3B1*^WT^ and *SF3B1*^R625/K666^ cell lines demonstrates that the deregulated splice pattern strictly depends on *SF3B1* status and on the 3’ss-sequence context. SF3B1^WT^ knockdown or overexpression do not reproduce the *SF3B1*^R625/K666^ splice pattern, qualifying *SF3B1*^R625/K666^ as change-of-function mutants. Mutagenesis of predicted branchpoints reveals that the *SF3B1*^R625/K666^-promoted splice pattern is a direct result of alternative branchpoint usage. Altogether, this study provides a better understanding of the mechanisms underlying splicing alterations induced by mutant SF3B1 in cancer, and reveals a role for alternative branchpoints in disease.

## Abstract (web summary)

Mutations in the splicing factor *SF3B1* are found in uveal melanoma. Here, Alsafadi *et al*. use RNA-sequencing data from these cancers and experimental models, and show that mutant SF3B1 promotes alternative branchpoints in a specific gene subset and that the mutant protein gains a new function.

## Graphical abstract

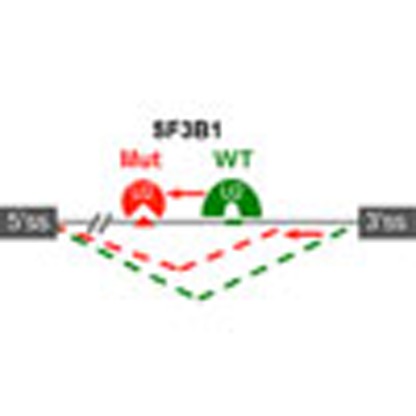

Discovery of recurrent missense mutations in splicing factors in cancers revealed the importance of the spliceosome pathway as a direct actor in carcinogenesis and questioned functional roles and molecular mechanisms of these mutations. *SF3B1* (*Splicing Factor 3B Subunit 1A*) encodes for a core component of the U2 small nuclear ribonucleoprotein (snRNP) complex of the spliceosome and is involved in early stages of splicing. Alterations in *SF3B1* were initially discovered in myelodysplastic syndromes (MDSs) and chronic lymphocytic leukemia (CLL), together with other mutations of splicing factors, such as *U2AF1*, *SRSF2* and *ZRSR2* (refs 1, 2, 3). Importantly, these genes encode proteins that are all involved in 3′-splice site recognition during RNA splicing^4^. It has been shown that *SF3B1* is mutated in a significant proportion (∼20%) of uveal melanoma (UM), a rare malignant entity deriving from melanocytes from the uveal tract^5^,^6^,^7^, and in other solid tumours at lesser frequencies^8^,^9^.

RNA splicing is a fundamental process in eukaryotes, which is carried out by the splicing machinery (spliceosome) composed of five snRNPs and additional proteins^10^. Introns contain consensus sequences that define the 5′ donor splice site (5′ss), branchpoint (BP) and 3′ acceptor splice site (3′ss), which are initially recognized by the U1 snRNP, SF1 protein and U2AF, respectively. U2AF is a heterodimer composed of U2AF2 (also known as U2AF65) and U2AF1 (also known as U2AF35), which recognize the poly-pyrimidine tract and the well-conserved AG dinucleotide sequence of 3′ss, respectively. After binding to the 3′ss, U2AF facilitates replacement of SF1 by U2 snRNP at the BP. Interaction between U1 and U2 snRNPs then triggers transesterification joining the 5′-end of the intron to the BP, most generally an adenosine located in a loosely defined consensus ∼25 nucleotides upstream of the 3′ss. The 5′ss and 3′ss are then ligated together and the branched intron is discarded^10^.

SF3B1 mediates U2 snRNP recruitment to the BP by interacting with the intronic RNA on both sides of the BP and with U2AF^11^. Structurally, the SF3B1 protein has an N-terminal hydrophilic region containing U2AF-binding motif and a C-terminal region, which consists of 22 non-identical HEAT (Huntingtin, Elongation factor 3, protein phosphatase 2A, Targets of rapamycin 1) repeats. Cancer-associated mutations in *SF3B1* are missense mutations with three major hotspots targeting the fifth, sixth and seventh HEAT repeats at codon positions R625, K666 and K700, respectively. Interestingly, K700 mutations are by far the most frequent in haematopoietic malignancies, whereas R625 mutations are prevailing in UM. These alterations affect residues that are predicted to be spatially close to one another and therefore might have a similar functional impact^1^.

Recently, RNA-sequencing (RNA-Seq) analysis of CLL, breast cancer and UM showed that a global splicing defect in *SF3B1*-mutated tumours consists in usage of cryptic 3′ss (hereafter called AG′) located 10 to 30 bases upstream of normal 3′ss, yet the underlying mechanism has remained poorly understood. It has been proposed that AG′ is located at the end of a sterically protected region in a specific region downstream the BP. Yet, not every potentially located AG′ was used in an *SF3B1*^MUT^ context^12^.

In the present study, RNA-Seq analysis of 74 primary UMs, mutated or not for *SF3B1,* confirmed the *SF3B1*^*MUT*^-promoted pattern identified by DeBoever *et al*.^12^, demonstrating the robustness of the deregulated pattern. By constructing *in cellulo* models, we show that *SF3B1*^*MUT*^ is the direct cause of the deregulated splice pattern and could be qualified neither as gain-of-function (that is, hyperactivity) nor loss-of-function, but rather as change-of-function mutants. Our experiments provided evidence that (i) mutant SF3B1 preferentially recognizes alternative BPs upstream of the canonical sites and (ii) the alternative 3′ss used in a *SF3B1*^*MUT*^ context are less dependent on U2AF. We propose a model of the SF3B1^MUT^ dysfunctions that sheds new light on the mechanism of splicing dysregulation in cancer. In addition, our data reveal a currently under-appreciated role for recently described alternative branch points^13^ in alternative splicing and disease.

## Results

### *SF3B1* mutations promote upstream alternative acceptors

Following initial finding of recurrent mutations of *SF3B1* gene in UM^5^, we set up an independent consecutive series of UM to analyse the effect of *SF3B1* hotspot mutations. This series included 74 T2–T4 tumours of different histologic types (21 epithelioid cell, 18 spindle cell and 35 mixed cases) treated by primary enucleation. Thirty-eight cases (51%) subsequently developed metastases and 40 patients (54%) died. *SF3B1* mutations were found in 16 tumours affecting two hotspots p.R625 and p.K666 (Supplementary Table 1). No mutation of other genes coding for splicing factors was observed. SNP array analysis did not reveal any chromosome loss or gain in the region containing *SF3B1* (2q33.1). The overall mutation rate of 22% (16/74) is comparable to the rate (19%) recently reported for *SF3B1* mutations in UM^5^,^6^,^7^.

**Figure 1:**
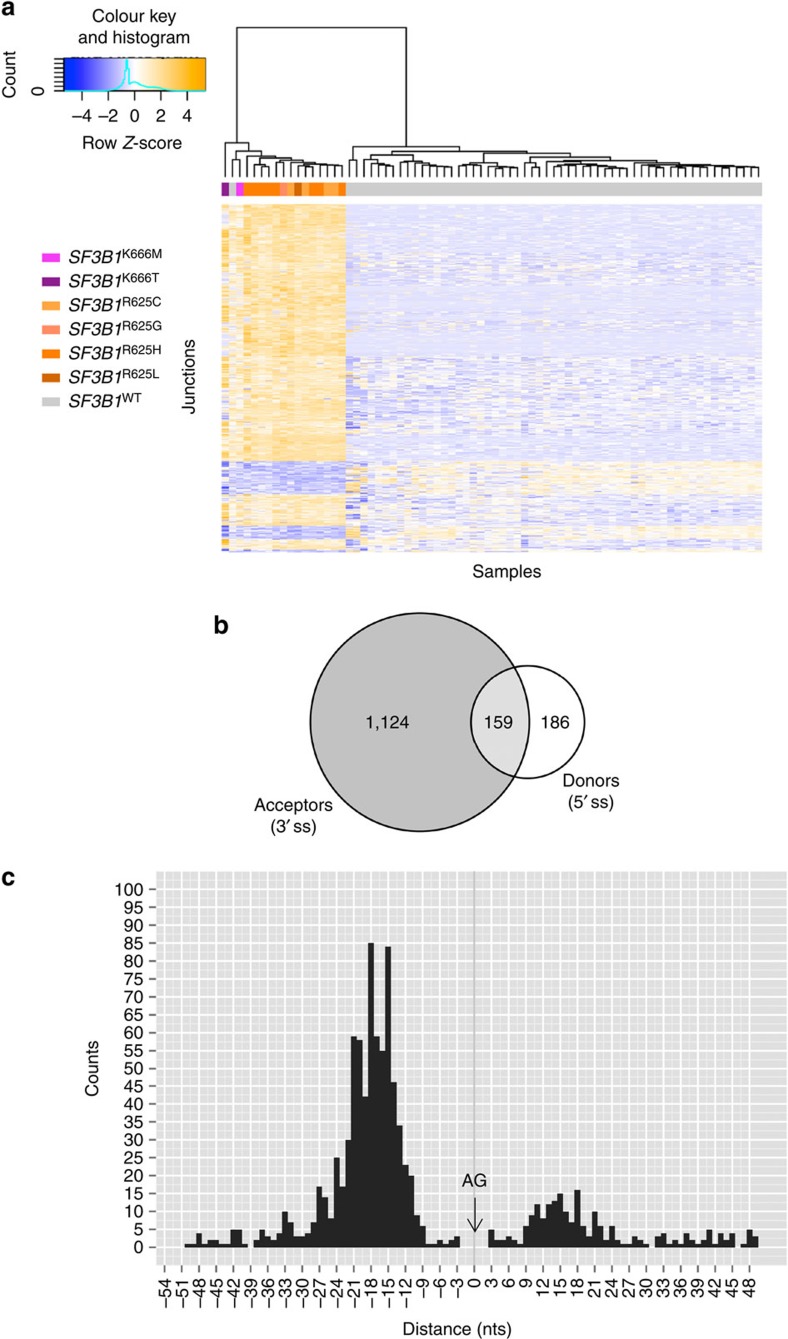
Differential splice junctions in *SF3B1*^MUT^ tumours. (**a**) Hierarchical clustering and heat-map analysis of differential splice junctions in tumour samples. The colours of squares below the tree denote the subtype of each sample. Below the array tree and the subtype identification row, the heat map of the 1,469 splice junctions is shown. The complete list of up- and downregulated splice junctions can be found in Supplementary Data 1. (**b**) Venn diagram of differential splice junctions in *SF3B1*^MUT^ compared with *SF3B1*^WT^ tumours. Numbers show the count of alterations within only 5′ss (186 events) or only 3′ss (1,124 events). The overlapping area represents junctions that are either ambiguously attributed to an alternative donor or acceptor site, or attributed to both alternative 3′ and 5′ splice sites (159 events). (**c**) Distances between the alternative and canonical *SF3B1*^MUT^-sensitive 3′ss. For alternative 3′ss within the 50 nts preceding the canonical 3′ss (765), the distance between the alternative (AG’) and corresponding canonical (AG) 3′ss was plotted as a histogram. Negative distances mean the alternative AG’ upstream of the canonical AG, whereas positive distances mean the AG’ downstream. The 0 point demarks the position of the canonical AG.

To evaluate the effects of *SF3B1* mutations on splicing, we performed transcriptome analysis of the UM cohort using RNA-Seq technique. Differential analysis of splice junctions between the *SF3B1*^MUT^ (*n*=16) and *SF3B1*^WT^ (*n*=56) tumours using DESeq2 (ref. 14) revealed an overall high level of differences. The top 1,469 differentially spliced junctions with *P*-values ≤10^−5^ (Benjamini-Hochberg) and absolute Log_2_(fold change)≥1 were selected for further analyses (Supplementary Fig. 1 and Supplementary Data 1). A hierarchical clustering of the 74 tumours using the 1,469 differential splicing junctions showed coherent changes in *SF3B1*^MUT^ tumours (Fig. 1a). A single *SF3B1*^WT^ tumour clustered together with *SF3B1*^MUT^ cases. Manual reanalysis excluded any variant of its *SF3B1-*coding sequence or any over- or under-expression of the *SF3B1* transcript and exome sequencing of this case failed to identify any mutation of the spliceosome genes as a potential genocopy.

Interestingly, 72% (1,060/1,469) of differential splice variants had no Ensembl Transcript identifiers (ENST) and these novel splice variants were found almost exclusively in *SF3B1* mutants. To be noticed, only 9% (12,866/142,458) of non-differential splice variants had no ENST. The acceptor splice site (3′ss) was altered in 1,124 differential junctions (76.5%), whereas the altered donor splice site (5′ss) was observed in 186 differential junctions (12.7%; Fig. 1b). For 159 junctions (10.8%), the novel junction was either ambiguously attributed to the alternative 5′ or 3′ss, or attributed to both alternative 5′ss and 3′ss.

The analysis of distances between the alternative and canonical *SF3B1*^MUT^-sensitive 3’ss showed repetitive peaks of alternative 3’ss every three nucleotides (Fig. 1c). Such spacing of two nucleotides suggests that frameshift variants are targeted by nonsense-mediated mRNA decay.

We observed that the majority (765 out of 1,124) of the *SF3B1*^MUT^-promoted alternative 3′ss—thereafter named AG′—were located within 50 nucleotides (nts) that precede the canonical 3′ss—thereafter named AG—with a clear clustering in the −12 to −24 nt region upstream of the canonical AG (Fig. 1c). No ENST Identifier exists for 675 out of these 765 AG′ alternative acceptor sites (88%).

These results are concordant with a recent study based on RNA-Seq data from CLL, breast cancer and UM samples^12^. DeBoever *et al*. showed that 619 cryptic 3′ss clustering 10–30 nucleotides upstream of canonical 3′ss were used in cancers with *SF3B1* mutations. Interestingly, we found 327 out of these 619 cryptic 3′ss (53%) to be differentially expressed in our data set, demonstrating the robustness of this splicing pattern despite the differences in the series of analysed tumours and bioinformatics pipelines.

### Sequence context determines acceptor sensitivity to *SF3B1* ^MUT^

To validate the splice pattern detected in *SF3B1*^MUT^ tumours and to determine if it is conferred by sequences in the region of the 3′ss, we performed minigene splicing assay. We selected seven 3′ss within the top differential splice junctions associated with *SF3B1*^MUT^ tumours (named as sensitive 3′ss) and two 3′ss found unaltered in an *SF3B1*^MUT^ context (named as insensitive 3′ss; Table 1). Regions containing the selected 3′ss were cloned into an ExonTrap vector and expressed into human cell lines with different *SF3B1* status: 2 UM cell lines, MP41 (*SF3B1*^WT^) and Mel202 (*SF3B1*^R625G^; Supplementary Figs 2 and 3). In addition, to directly determine whether the splice pattern detected in *SF3B1*^MUT^ tumours is dependent on *SF3B1* status, we used two isogenic HEK293T cell lines, *SF3B1*^WT^ and *SF3B1*^K666T^ obtained by the CRISPR/Cas9 technology. RNA-Seq data, as visualized by IGV (Integrative Genomics Viewer), showed different mutation rates of 30% in Mel202 cells and 14% in *SF3B1*^K666T^ HEK293T cells, because of multiple copies of the wild-type *SF3B1* allele in these aneuploid cell lines. As expected, Mel202 (*SF3B1*^MUT^) clusters with the *SF3B1*^MUT^ tumours and MP41 (*SF3B1*^WT^) clusters with *SF3B1*^WT^ tumours. Probably due to its low level of *SF3B1*^K666T^ expression, *SF3B1*^K666T^-HEK293T clusters together with HEK293T (WT) and *SF3B1*^WT^ tumours (Supplementary Fig. 2).

**Figure 2:**
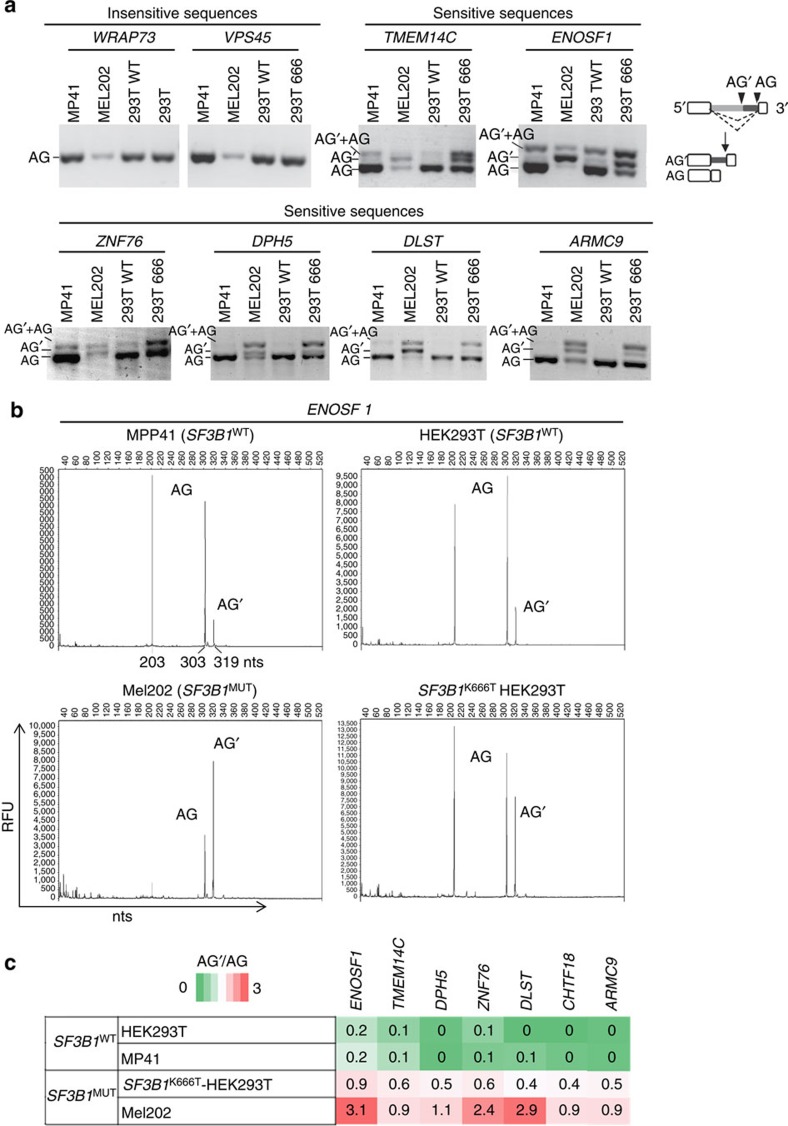
*In cellulo* validation of differential splice junctions. (**a**) Minigene splice assay of two *SF3B1*^MUT^-insensitive 3′ss (*WRAP73* and *VPS45*) and 6 *SF3B1*^MUT^-sensitive 3′ss (*TMEM14C, ENOSF1, ZNF76, DPH5, DLST, ARMC9*). Gel electrophoresis shows the different splicing processes for minigene ExonTrap constructions in *SF3B1*^WT^ cell lines (MP41 and HEK293T) and *SF3B1*^MUT^ cell lines (Mel202 and K666T-*SF3B1* HEK293T). The lower band corresponds to the variant generated by the usage of the canonical 3′ss (AG). The intermediate band corresponds to a splice generated by the usage of the alternative 3′ss (AG’). The upper band is the heteroduplex formation from two bands (AG and AG’). (**b**) Analysis of alternative AG’ and canonical AG usage of the ExonTrap construct (*ENOSF1*) in cell lines by capillary electrophoresis of RT–PCR products. Representative GeneMarker electrophoregrams for fragment analysis of *ENOSF1* minigene cDNA expression are shown. The *x* axis represents molecular size (in nucleotides (nts)) of PCR products, and the *y* axis indicates relative fluorescent units (RFUs). The peak of 203 nts refers to the internal splicing of the pET01 ExonTrap vector using its 3′ss and 5′ss. The peak of 303 nts corresponds to the usage of the canonical AG, whereas the peak of 319 nts corresponds to the usage of alternative AG’ WT. (**c**) Heat-map analysis using the AG’/AG ratio of the top differential splice junctions in cell lines as determined by capillary electrophoresis of RT–PCR products.

The splice forms corresponding to canonical AG usage were found expressed after transfection of the insensitive 3′ss constructs in all cell lines, regardless of their *SF3B1* status (Fig. 2a). Likewise, for the seven sensitive 3′ss constructs, the splice forms corresponding to canonical AG usage were found expressed in the *SF3B1*^WT^ cell lines MP41 and HEK293T. By contrast, the *SF3B1*^MUT^ cell lines Mel202 and *SF3B1*^K666T^ HEK293T expressed the alternative splice forms using the alternative AG′ in addition to the canonical AG (Fig. 2a). The correspondence between band sizes and splice usage was verified by Sanger sequencing. Interestingly, the ratio of the alternative AG′ versus canonical AG usage (AG′/AG) based on capillary electrophoresis profiles varied according to the *SF3B1*^MUT^*/SF3B1*^WT^ rate in the cell lines (Fig. 2b,c). Of note, a faint but significant usage of alternative AG′ in *SF3B1*^WT^ cell lines was detected on the capillary electrophoresis profiles for three sensitive 3′ss, *ENOSF1*, *TMEM14C* and *ZNF76* (AG′/AG in *SF3B1*^WT^ cell lines=0.2, 0.1 and 0.07, respectively), implying that the AG′ usage may be reinforced rather than induced *de novo* in an *SF3B1*^MUT^ context (Fig. 2b).

In conclusion, we demonstrate that the aberrant splice pattern is strictly dependent on the *SF3B1*^MUT^ status and on sequences in the close vicinity of the sensitive 3′ss.

### *SF3B1* hotspot mutations are change-of-function mutations

**Figure 3:**
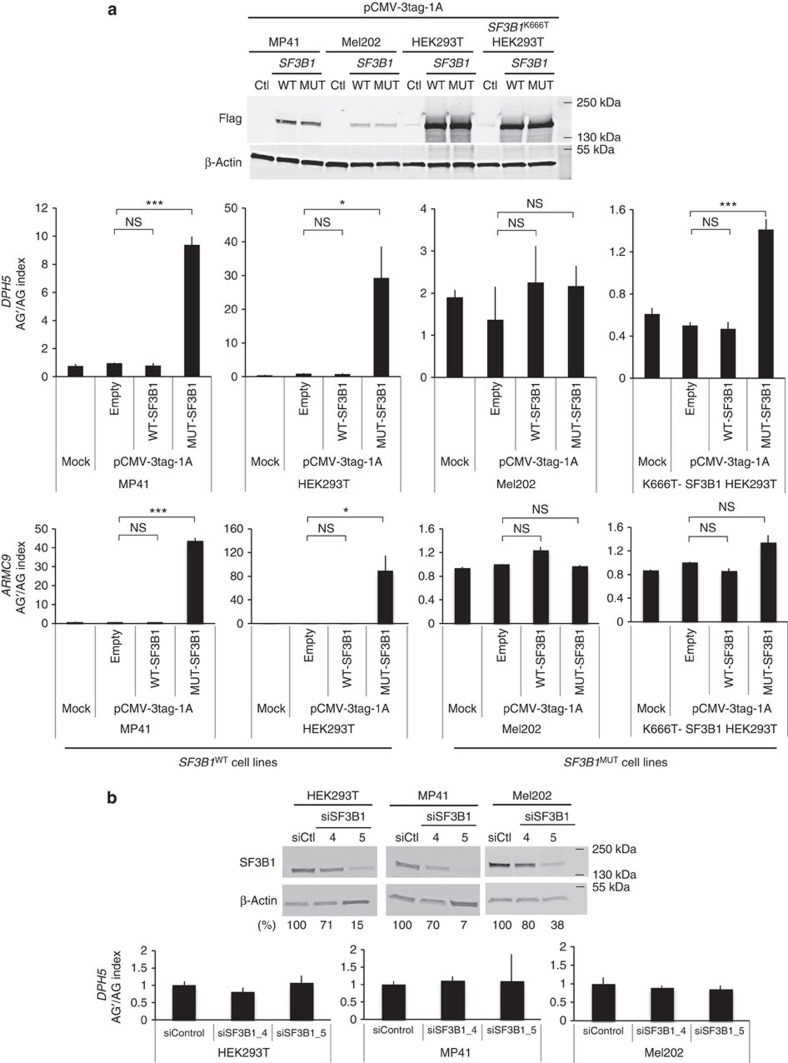
Overexpression and underexpression of wild-type *SF3B1* do not reproduce the splice pattern of *SF3B1* hotspot mutations. (**a**) Effect of overexpression of wild-type and mutated *SF3B1* on the AG’/AG ratio of *DPH5* and *ARMC9* in *SF3B1*^WT^ and *SF3B1*^MUT^ cell lines. MP41, Mel202, HEK293T and *SF3B1*^K666T^-HEK293T cell lines were transiently transfected with expression vectors for *SF3B1*^WT^ and *SF3B1*^K700E^. The protein overexpression was confirmed by immunoblotting with anti-Flag using β-actin as a loading control (upper panel). Ratios of expression levels of alternative AG’ and canonical AG forms (AG’/AG) of *DPH5* and *ARMC9* were determined by quantitative RT–PCR (lower panel). The results are average of three replicates and are represented as mean±s.d. Paired *t*-test was used to generate the *P*-values comparing each condition to the empty vector transfection: NS, non-significant; *P>0.05; ***P<0.001. (**b**) Effect of siRNA-mediated knockdown of *SF3B1* on the AG’/AG ratio in cell lines. HEK293T, MP41 and Mel202 cells were transiently transfected with non-target control siRNA or two different si*SF3B1*: ‘4’and ‘5’. Proteins and RNA were extracted at 48 h after transfection. siRNA-mediated knockdown was confirmed by immunoblotting with anti-SF3B1, using anti-β-actin as a loading control. Numbers represent the protein band intensity normalized to β-actin and expressed as a percentage of control samples (upper panel). Ratios of expression levels of alternative AG’ form to the expression level of canonical AG form (AG’/AG) of *DPH5* were determined by quantitative RT–PCR (lower panel). The results are average of three replicates and are represented as mean±s.d.

The mode of action of *SF3B1* mutant was then addressed by analysing endogenous *DPH5* and *ARMC9* transcripts. The different cell lines were transiently transfected with expression vectors for *SF3B1*^WT^ and *SF3B1*^K700E^ and examined 48 h later for the AG′/AG usage of endogenous 3′ss (Fig. 3a). We represent the shift from the canonical AG to the alternative AG′ by the AG′/AG index, which is the ratio of mRNA expression of AG′ form to AG form of a validated gene, *DPH5* or *ARMC9*, determined by quantitative reverse transcription (RT)–PCR. The overexpression of *SF3B1*^K700E^ significantly increased the AG′/AG index in *SF3B1*^WT^ cell lines (10- and 32-fold increases for *DPH5* in MP41 and HEK293T, respectively), whereas overexpression of *SF3B1*^WT^ had no effect on the AG′/AG index. The overexpression of *SF3B1*^K700E^ increased by only three-fold the AG′/AG index in *SF3B1*^K666T^ HEK293T (transcript mutation rate=14%) and did not modify it in Mel202 cell line (transcript mutation rate=30%), which may indicate a saturating effect of *SF3B1*^MUT^. Similar results were obtained with the endogenous sensitive 3′ss of *ARMC9*. We conclude that *SF3B1* mutation does not lead to a hyper-activity of the protein, as its phenotype is not reproduced by *SF3B1* overexpression.

To determine whether *SF3B1* mutations are loss-of-function mutations, we then assessed the effect of *SF3B1* short interfering RNA (siRNA)-mediated knockdown on alternative splicing in *SF3B1*^WT^ HEK293T and MP41 and in *SF3B1*^MUT^ Mel202. Non-target siRNA was used as a negative control and siRNA-mediated knockdown was confirmed by immunoblotting (Fig. 3b). As shown in Fig. 3b, *SF3B1* siRNA-mediated knockdown did not have any significant effect on AG′/AG index despite up to 93% of SF3B1 protein level reduction. These findings demonstrate that the *SF3B1*^MUT^ splice pattern is not mimicked by *SF3B1* knockdown.

Altogether, our results provide the first evidence that *SF3B1* mutants are neither gain- (hyperactive) nor loss-of-function mutants, and suggest change-of-function consequences.

### *SF3B1* ^MUT^-promoted AG′ are weakly dependent on U2AF

**Figure 4:**
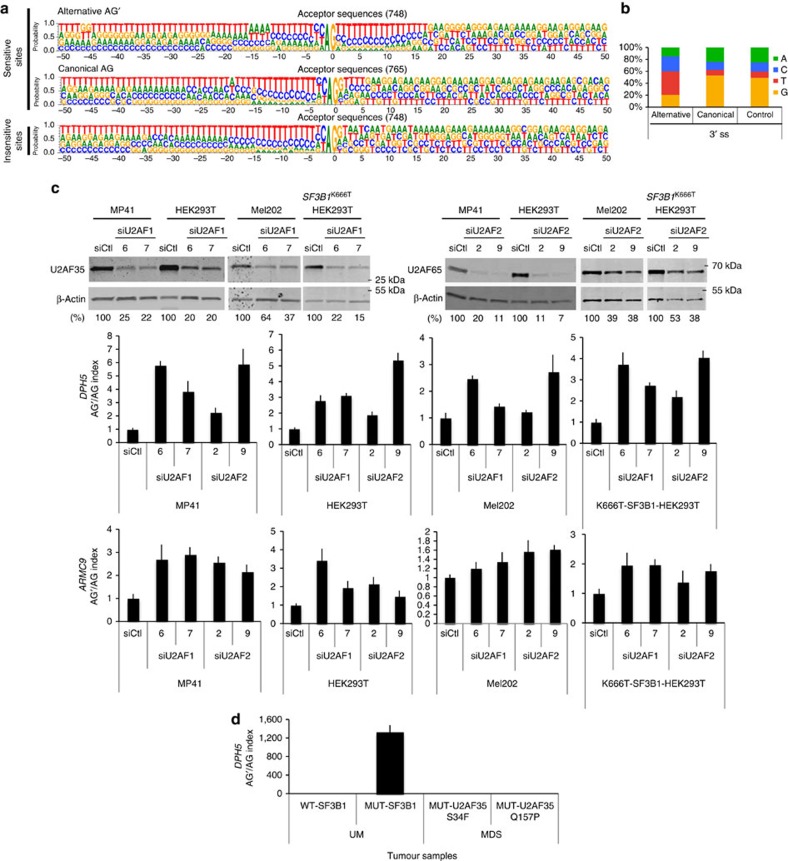
Characterization of alternative 3′ss (AG’) sequences. (**a**) Comparison of sequence logos of 3′ss sensitive to SF3B1 status with canonical (AG) and alternative (AG’) sequences and 3′ss insensitive to SF3B1 status. One-hundred-nucleotide-long sequences surrounding the 3′ss were used to generate sequence logos with WebLogo. The height of each letter indicates the preference strength for that nucleotide at each position. (**b**) The proportion of the nucleotides immediately following the alternative (AG’), the canonical (AG) and insensitive 3′ss. (**c**) Effect of *U2AF35* and *U2AF65* siRNA-mediated knockdown on the AG’/AG ratio in cell lines. MP41 and HEK293T (*SF3B1*^WT^) and Mel202 and *SF3B1*^K666T^-HEK293T (*SF3B1*^MUT^) cells were transiently transfected with non-target control siRNA, si*U2AF35* or si*U2AF65*. Proteins and RNA were extracted at 48 h after transfection. siRNA-mediated knockdown was confirmed by immunoblotting with anti-U2AF35 and anti-U2AF65, using anti-β*-*actin as a loading control. Numbers represent the protein band intensity normalized to β*-*actin and expressed as percentage of control samples (upper panel). Ratio of expression levels of alternative AG’ form to the expression level of canonical AG form (AG’/AG) of *DPH5* and *ARMC9* was determined by quantitative RT–PCR (lower panel). The results are average of three replicates and are represented as mean±s.d. (**d**) Effect of *U2AF35* hotspot mutations on the AG’/AG ratio of *DPH5* in MDS tumours. Ratio of expression levels of alternative AG’ form to the expression level of canonical AG form (AG′/AG) of *DPH5* was determined by quantitative RT–PCR in two MDS samples, each harbouring one of the two *U2AF35* hotspot mutations, S34F and Q157P and compared with mutated and wild-type *SF3B1* uveal melanoma (UM) samples.

As sensitivity to *SF3B1* mutants was conferred by sequences in the close vicinity of 3′ss (Fig. 2), we searched for a sequence pattern associated with sensitive 3′ss. We compared the sequences of alternative AG′, corresponding canonical AG, and insensitive 3′ss (Fig. 4a). Two obvious features were found associated with AG′ consensus sequence: the paucity of G nucleotide at +1 position, and the frequency of A nucleotides at −11 to −14. Only 20% of G was observed for the alternative AG′, compared with ∼50% of G for both canonical AG and insensitive 3′ss (Fig. 4b). The high proportion of AG-(C/T) in the alternative AG′ site (65% versus 23% in AG′ and AG, respectively) is best explained by the presence of the AG′ within the polypyrimidine tract of the corresponding canonical AG. AG-G is part of the recognition motif of U2AF1 (ref. 15). The low frequency of G nucleotide immediately following alternative AG′ sites suggests lesser dependence to U2AF1 compared with the canonical AG at the step of 3’ss recognition^16^,^17^,^18^. To test this hypothesis, MP41 and HEK293T cells were transiently transfected with si*U2AF1* or si*U2AF2*. Efficient knockdown was confirmed by immunoblotting. As shown in Fig. 4c, both *U2AF1* and *U2AF2* knockdowns increased the AG′/AG index of *DPH5* and *ARMC9* in *SF3B1*^WT^ cells, and had no significant effect in *SF3B1*^R625/K666^ cells (Fig. 4c). These findings suggest that AG′ is less dependent on U2AF than the competing canonical AG.

One hypothesis to explain why U2AF knockdown partially mimicked the effect of *SF3B1*^MUT^ was that the SF3B1–U2AF2 interaction^11^ might be decreased in the case of mutant SF3B1. However, U2AF2 and U2AF1 antibodies immunoprecipitated equally SF3B1^WT^ and SF3B1^K700M^, implying no detectable alteration in the SF3B1^MUT^–U2AF interaction (Supplementary Fig. 4).

Considering that *U2AF1* mutations are reported in ∼10% of patients with MDS and associated with partial functional impairment in regulated splicing^2^,^19^, we tested two MDS samples each harbouring one of the two *U2AF1* hotspot mutations, S34F and Q157P. Neither of these MDS samples presented any increase of the AG′/AG index of *DPH5*, demonstrating that *U2AF1* and *SF3B1* hotspot mutations do not lead to the same aberrant splicing phenotype (Fig. 4d).

Overall, our results exclude a defective SF3B1^MUT^–U2AF interaction and show that *U2AF1*^MUT^ and *SF3B1*^MUT^ can induce different splicing patterns. Importantly, the rarity of G at the position +1 after AG′ as well as the increase of AG′/AG transcript ratio when U2AF is depleted suggest that *SF3B1*^MUT^-promoted 3′ss (AG′) is less dependent on U2AF as compared with downstream canonical 3′ss (AG).

### Alternative BP usage in an *SF3B1* ^MUT^ context

The second feature characterizing the AG′ consensus sequence is the presence of frequent adenosines at a distance of 11–14 nts preceding the AG′, which could represent alternative BPs (Fig. 4a). Exploring this hypothesis, we investigated whether *SF3B1*^MUT^ alters BP choice.

**Figure 5:**
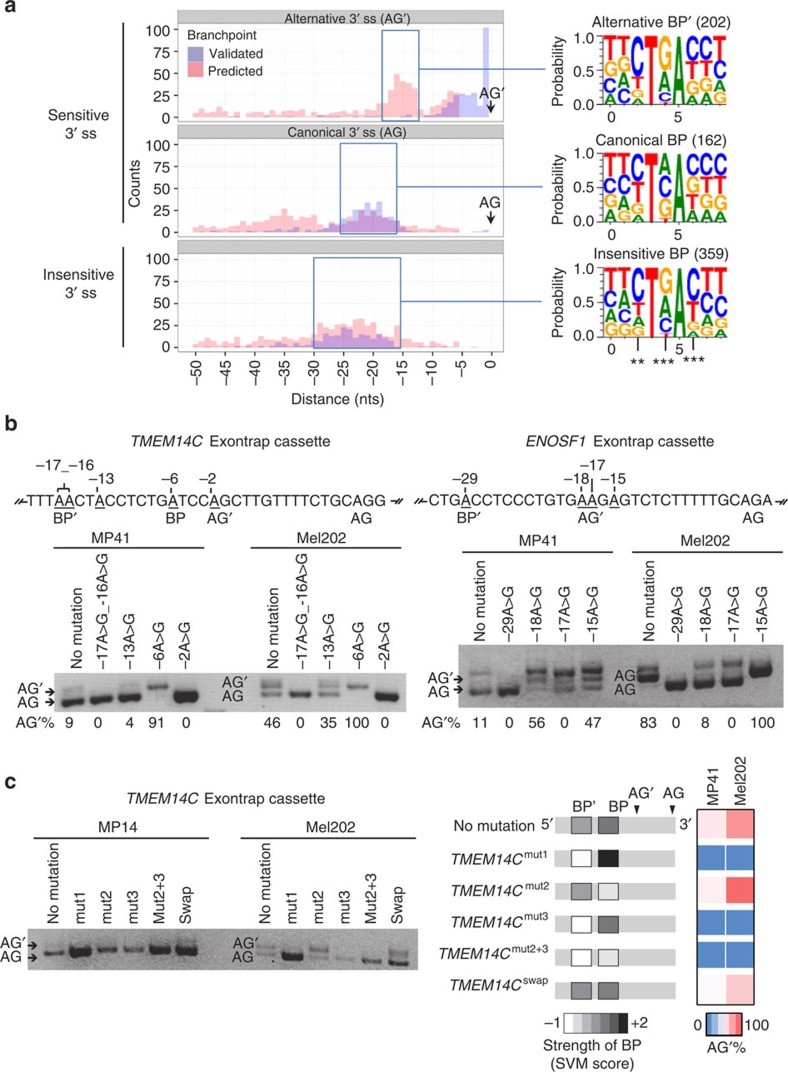
Identification of alternative branchpoint usage in an *SF3B1*^MUT^ context. (**a**) Analysis of distances between the branchpoints and the associated alternative (AG’), canonical (AG) or insensitive 3′ss. Left panel: Zero represents the position of the AG’, AG or insensitive 3′ss. Red bars represent the clusters of branchpoints predicted by the tool of SVM, blue bars represent the experimentally determined BP data set reported by Mercer *et al*.^13^. Right panel: the extracted branchpoint sequence logos generated by WebLogo. **Significantly different in the three branchpoint patterns (*χ*^2^ test, *P*<0.01), ***Significantly different in the three branchpoint patterns (*χ*^2^ test, *P*<0.001). (**b**) Point mutations of branchpoints in *TMEM14C* and *ENOSF1* ExonTrap constructs. All adenosines within a region of 30 nts preceding the canonical AG were mutated into guanines. Mutant constructs were expressed in MP41 (*SF3B1*^*WT*^) and Mel202 *(SF3B1*^*MUT*^) cells followed by RT–qPCR. The lower band corresponds to the variant generated by the usage of the canonical 3′ss (AG). The intermediate band corresponds to the variant generated by the usage of the alternative 3′ss (AG’). The upper band is the heteroduplex formation from two bands (AG and AG′). The numbers represent the ratio of AG’ usage as determined by capillary electrophoresis. (**c**) Base-pairing potential mutants of *TMEM14C*. Mutant constructs (sequences shown in Supplementary Fig. 5) were expressed in MP41 (*SF3B1*^WT^) and Mel202 (*SF3B1*^MUT^) cells followed by RT–qPCR. The lower band corresponds to the variant generated by the usage of the canonical 3′ss (AG). The upper band corresponds to the variant generated by the usage of the alternative 3′ss (AG’). A schematic presentation of the strength of the resulting branchpoints as estimated by their SVM score is shown on the right panel. The ratio of AG’ usage as determined by capillary electrophoresis in MP41 and Mel202 cells is shown as a heat map.

We used the online tools SVM (Support Vector Machine algorithm)-BPfinder and the Human Splicing Finder to predict the BP of the 744 sensitive 3′ss. The predicted BP clustered together at ∼22 nts upstream the insensitive 3′ss. However, the predicted BP for the alternative and canonical 3′ss showed a bi-modal distribution centred at 5 and 15 nts upstream the AG′, and at 20 and 35 nts upstream AG (Fig. 5a). Using the experimentally determined BP data set recently reported by Mercer *et al*.^13^, we found 286 out of the 744 sensitive 3’ss with a determined BP in an *SF3B1*^*WT*^ context (Fig. 5a). Remarkably, we found that 37% (105/286) of the A of the BP coincided with the A of AG′. Most of the other BP were closely distributed upstream of AG′ at an average of 5 nts. This superimposition of BP and AG′ made unlikely the usage of the same BP for both the canonical AG and alternative AG′. We thus suspected the usage of alternative branchpoint (BP′), possibly corresponding to the second peak of predicted BP around 35 nts 5′ to the AG and ∼15 nts 5′ to the AG′ (Fig. 5a).

To explore such hypothesis, we mutated all adenosines within a region of 30 nts preceding the canonical AG in two sensitive sequences, *TMEM14C* and *ENOSF1*. These two minigenes were selected for their low frequency of adenosines upstream the 3′ss in order to limit the number of required site-directed mutations. We then expressed these variant acceptor sequences in MP41 (*SF3B1*^*WT*^) and Mel202 *(SF3B1*^*MUT*^) cells followed by RT–quantitative PCR and fragment analysis by capillary electrophoresis as described above (Fig. 5b).

The observed consequences of *TMEM14C* mutants were the following: (i) the -17A>G_-16A>G-double mutation completely abolished the usage of the alternative AG′ in both recipient cell lines; (ii) the -13A>G mutation had no consequence on 3′ss usage; (iii) the -6A>G mutation completely abolished the usage of the canonical AG; (iv) the -2A>G mutation completely abolished the usage of the alternative AG′ as it destroyed the AG′ site.

We interpret these data as indicating that the A at 30 nts upstream of AG is the BP′ for AG′, as its mutation allowed only the use of the canonical AG regardless of *SF3B1* status. Alternatively, -6A>G mutation switched the usage of the AG to the usage of AG′, arguing that this site may serve as the BP for the canonical AG. Our data therefore support the existence of two branchpoints, BP and BP′, differentially used depending on the SF3B1 status.

Similar observations were obtained with the *ENOSF1* construct confirming the existence of two different BPs. Specifically, we could determine the BP′ of AG′ at 29 nts preceding the AG (-29A>G). The -18A>G mutation disturbed the usage of both AG′ and AG, whereas the -17A>G mutation disturbed AG usage and inhibited the usage of AG′. In fact, the later mutation created another alternative acceptor site replacing the AG′ (AAG>AGG), and competing with the canonical AG. The potential BP was loosely defined for *ENOSF1*, because of the multiple adenosines in the vicinity of AG′ participating to the usage of the canonical AG. To be noticed, the -15A>G mutation of the nucleotide immediately following the AG′ strengthened the alternative site, presumably by reinforcing the binding of U2AF1 to the AG′-G site. Thus, the analyses of both the *TMEM14C* and *ENOSF1* genes indicate that *SF3B1*^MUT^ affects the 3′ss choice by promoting the use of alternative BPs.

SF3B1 plays a major role in U2 snRNP recruitment to the BP. To determine whether the potential of BP sequences to form base-pairing interaction with U2 snRNA (small nuclear RNAs) can modulate the sensitivity to *SF3B1* mutations, BP and BP′ mutants of *TMEM14C* were generated (Supplementary Fig. 5). The strength of the resulting BPs was estimated by their SVM score^20^ (Fig. 5c). The *TMEM14C*^mut1^ allows a perfect base-pairing of BP with U2 snRNA^21^; *TMEM14C*^mut2^ contains a suboptimal BP; *TMEM14C*^mut3^ contains a defective alternative BP′; *TMEM14C*^mut2+3^ includes both *TMEM14C*^mut2^ and *TMEM14C*^mut3^ mutated regions and *TMEM14C*^*swap*^ contains swapped endogenous BP and BP′ sequences.

The consequences of these mutants were then assessed by the AG′/AG index (RT–PCR). Enhancing the base-pairing of the BP region (*TMEM14C*^mut1^), disrupting BP′ (*TMEM14C*^mut3^) or combining a disrupted BP′ with a suboptimal BP (*TMEM14C*^mut2+3^) led to a total inhibition of AG′ usage, regardless of the *SF3B1* status. Decreasing the strength of BP (*TMEM14C*^mut2^) led to a reinforcement of AG′ usage (Fig. 5c). Interestingly, swapping the BP and BP′ sequences (*TMEM14C*^swap^) decreased AG′ usage, which could be interpreted as a higher strength of BP′ as compared with BP to form base-pairing interactions with U2 snRNA.

We extended this finding by an *in silico* comparison of the sequence patterns of alternative, canonical and insensitive BPs. We show that the canonical, alternative and insensitive BP presented distinct patterns (Fig. 5a), with significant sequence differences at positions +2, +4 and +6 of the motif (+5 being the A of the BP).

These data suggest that *SF3B1*^MUT^ favours the use of BP′ with stronger base-pairing potential with U2 snRNA compared with the downstream BP.

Collectively, in an *SF3B1*^MUT^ context, the stronger affinity of BP′ for U2 snRNA when compared with BP may allow the use BP′ with suboptimal AG′ (not followed by G, Fig. 4b) and may explain the lower dependence of AG′ on U2AF (Fig. 4c).

## Discussion

Here, we addressed the consequences of *SF3B1* hotspot mutations on splicing in UM and its underlying mechanisms. First, we observed that *SF3B1* hotspot mutations in UM are associated with deregulation of a restricted subset (∼0.5%) of splice junctions, mostly caused by the usage of alternative 3′ss (AG′) upstream of the canonical 3′ss (AG). This finding is concordant with a recent publication^12^, implying the robustness of the deregulated splice pattern in *SF3B1*^MUT^ tumours. Furthermore, this pattern is shared by tumours having for origin different cell lineages^12^,^22^. Second, we show here that *SF3B1*^MUT^ pattern was reproduced neither by knockdown nor by overexpressing wild-type SF3B1, indicating that *SF3B1* mutants could be qualified as change-of-function mutants. Third, and important, our data provide significant progress in understanding the molecular mechanisms underlying alternative 3′ss regulation by *SF3B1*^MUT^. We show that this mechanism involves a misregulation of BP′ usage, which have been largely overlooked in previous studies of alternative splicing and have been identified only recently on a large scale^13^.

Based on *in silico* data, DeBoever *et al*. proposed that *SF3B1*^MUT^-induced alternative 3′ss (AG′) is located at the end of a sterically protected region in a specific region downstream the canonical BP. Yet, not every potentially well-located AG′ was used in an *SF3B1*^MUT^ context, suggesting additional or different requirements for *SF3B1*^MUT^ selectivity^12^. They hypothesized no alternative BP usage as a mechanism of AG′ selection, because of the observed limited distances between AG and AG′. Strikingly, however, we showed here that mutagenesis of the predicted BP′ and the predicted canonical BP abrogated usage of AG′ and AG, respectively, confirming the existence of two BPs differentially used according to *SF3B1* status. Interestingly, since submission of our work, Darman *et al*. reported findings fully confirming our results. They also showed the consequences of *SF3B1* mutations on transcription through the generation of nonsense-mediated mRNA decay-sensitive aberrant spliced transcripts^23^.

Until recently, only few examples of alternative branchpoints were reported, such as in human *XPC* and rat fibronectin genes^24^,^25^. However in 2015, the genome-wide identification of BPs revealed that one-third (32%) of introns have at least two BPs^13^, but little is known about their regulation. Our findings provide the first evidence that misregulation of alternative BPs is involved in physiology or pathology.

**Figure 6:**
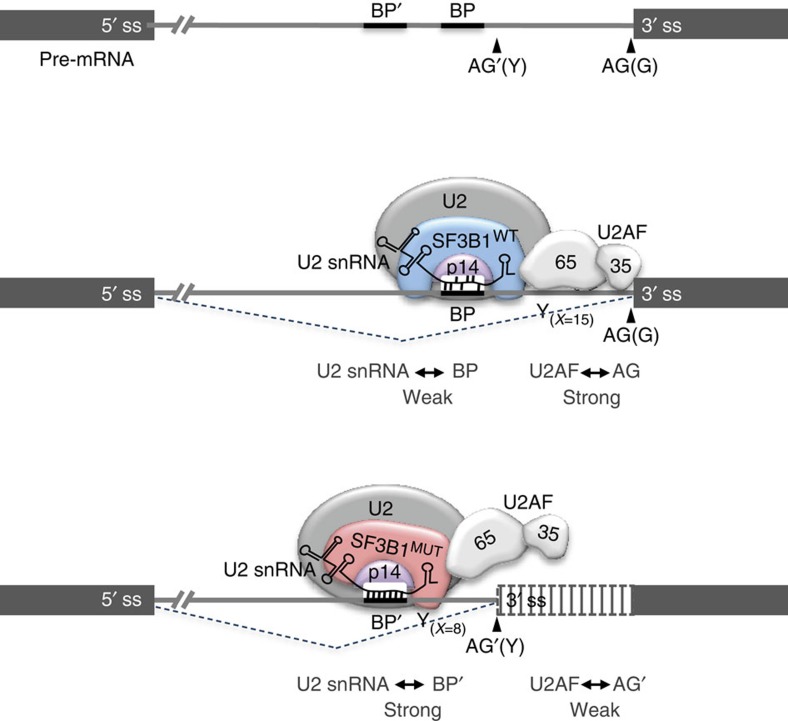
A model for alternative splicing dysregulation induced by *SF3B1* hotspot mutations. The 3′ss contains a segment, which is rich in pyrimidines (Y), a well-conserved AG dinucleotide and a branchpoint (BP) sequence recognized by the U2 snRNP. The U2 snRNP complex binds to the intron through base-pairing interactions between the BP sequence and the U2 snRNA, and through interactions between intron sequences, SF3B1 and p14. The HEAT repeats of SF3B1 form helical structures that occlude the surface of RNA recognition motif of p14. U2 snRNP containing SF3B1^WT^ recognizes the canonical U2AF-dependant BP. The hotspot mutations of *SF3B1* targeting the HEAT repeats occur on the inner surface of the structure and might induce a conformational change in the U2 snRNP complex altering its selectivity for BPs. U2 snRNP containing SF3B1^MUT^ has more stringent requirement for BP sequences and less for U2AF-dependent sequences, leading to the binding of alternative branchpoints (BP’) with high potential of base-pairing with U2 snRNP. AG, canonical 3′ss; AG’, alternative 3′ss; *x*, average number of pyrimidines; Y, pyrimidine.

Our findings indicate that *SF3B1*^MUT^-induced alternative 3′ss usage relies on three properties: an AG′ with lower affinity to U2AF than the canonical 3′ss, the presence of an BP′ with a higher affinity to U2 snRNA than canonical BP and the location of BP′ at a distance of 11–14 nts preceding the AG′. Based on these findings and on current understanding of SF3B1 function, we propose the following model for how *SF3B1*^MUT^ exerts its effects (Fig. 6). Because BP′ potential to form base-pairing interactions with U2 snRNA is generally superior to that of canonical BP, we suggest that U2 snRNP containing SF3B1^MUT^ has more stringent requirement for BP sequences than U2 snRNP-containing SF3B1^WT^. Consistently, the hotspot mutations of *SF3B1* target the HEAT repeats of SF3B1, which form helical structures that occlude the binding surface for RNA recognition motif of p14, a component of U2 snRNP that binds the BP^1^,^26^. The hotspot mutations of *SF3B1* in the HEAT repeats occur on the inner surface of the structure and may induce a conformational change in the U2 snRNP complex altering its selectivity for BPs. It is likely that stronger BP′ (in terms of U2 snRNA complementarity) can compensate for lower AG affinity to U2AF, leading to the recognition of BP′ in a U2AF-independent manner (or less dependent than in the case of canonical BP). This model is supported by our U2AF depletion experiments and is consistent with previous findings that BP recognition may depend or not on AG binding to U2AF35, according to BP and 3′ss sequence and organization^11^,^16^,^17^,^18^. In contrast, *SF3B1*^WT^ may allow a more promiscuous binding of U2 snRNA to both canonical and alternative BPs, and in this case, the final choice of BP may be determined by context, especially 3′ss affinity for U2AF.

Further work is required to evaluate the molecular mechanism by which the mutations of the SF3B1 HEAT domains may influence the base-pairing potential of U2 snRNA. The functional impact of *SF3B1*^MUT^-deregulated splicing pattern on oncogenesis also remains to be understood. Meanwhile, our study opens new possibilities for applying the deregulated splicing pattern as a screening tool as well as for targeting the splicing deregulation as a therapeutic strategy in UM and other *SF3B1*^*MUT*^-associated diseases^27^.

## Methods

### Patient cohort

A series of 109 consecutive patients diagnosed for UM without metastasis at diagnosis and treated by primary enucleation at the Institut Curie between January 2006 and December 2008 was assembled. RNA extracted from the tumour specimens was qualified for 74 (74/109) cases, which defined the patient cohort for this study.

RNA samples were obtained from surgical residual tumour tissues. In accordance to the national law on the protection of individuals taking part in biomedical research, patients were informed by their referring oncologist that their biological samples could be used for research purposes and they gave their verbal informed consent. All analyses done in this work were approved by the Institutional Review Board and Ethics Committee of the Institut Curie Hospital Group.

### DNA and RNA sequencing

Tumour DNA and RNA were provided by the Biological Resource Center of the Institut Curie. The DNA was extracted from frozen tumour or formalin-fixed paraffin-embedded samples using a standard phenol/chloroform procedure. *SF3B1* was sequenced by Sanger methods as previously described^5^. Primers used for Sanger sequencing are: (forward) 5′-CCAACTCATGACTGTCCTTTCT-3′ and 5′-TGGAAGGCCGAGAGATCATT-3′.

The total RNA was isolated from frozen tumour samples using a NucleoSpin Kit (Macherey-Nagel). cDNA synthesis was conducted with MuLV Reverse Transcriptase in accordance with the manufacturers’ instructions (Invitrogen), with quality assessments conducted on an Agilent 2100 Bioanalyzer. Libraries were constructed using the TruSeq Stranded mRNA Sample Preparation Kit (Illumina) and sequenced on an Illumina HiSeq 2500 platform using a 100-bp paired-end sequencing strategy. An average depth of global sequence coverage of 114 million and a median coverage of 112 million was attained.

### RNA-Seq analysis

TopHat (v2.0.6)^28^ was used to align the reads against the human reference genome Hg19 RefSeq (RNA sequences, GRCh37) downloaded from the UCSC Genome Browser (http://genome.ucsc.edu). Read counts for splicing junctions from junctions.bed TopHat output were considered. Differential analysis was performed on junction read counts using DESeq2 (ref. 14). Only alternative acceptor splice sites (two or more 3′ss with junctions to the same 5′ss) and alternative donor splice sites (two or more 5′ss with junctions to the same 3′ss) were considered for this analysis.

Fifty-nucleotide-long sequences surrounding the splice acceptor sites were extracted to generate sequence logos using WebLog 3 (http://weblogo.threeplusone.com/)^29^ with the default parameters, the classic colour scheme and the unit frequency being plotted as ‘probability’.

The data set supporting the results of this article is available on ArrayExpress repository under the accession E-MTAB-4097.

### BP sequence analysis

The online tools SVM-BPfinder^20^ (http://regulatorygenomics.upf.edu/Software/SVM_BP/) and the Human Splicing Finder^30^ (http://www.umd.be/HSF/) were used to predict the BPs. The SVM_BP code was altered to allow for BP six base pairs from the 3′ss by setting minidist3ss=6 in svm_getfeat.py.

### Minigene constructs

For each selected candidate gene alternative AG′-centred sequence of ∼200 nucleotides was PCR amplified from the genomic DNA of HEK293T cells using Phusion Hot Start II High Fidelity DNA Polymerase (Thermo Fisher Scientific). The primer sequence information is provided in Supplementary Table 2. We introduced 15 bases of homology with the ends of the linearized vector at the 5′-end of the forward and reverse primers. Using In-fusion HD cloning kit (Clontech), we cloned the amplicon into the *Bam*H1 site of pET01 ExonTrap vector (Mobitec) containing a functional splice donor site (Supplementary Fig. 3).

### Wild-type and mutated *SF3B1* constructs

A pCMV-3tag-1A vector containing wild-type *SF3B1* was synthesized by Genscript Corporation. Because mammalian *SF3B1* cDNA sequence was found unclonable in bacteria, a synthetic sequence was generated after codon-optimization for expression in bacteria. The full sequence of codon-optimized *SF3B1* is available upon request. K700E mutation in *SF3B1* was introduced using QuikChange II Site Directed Mutagenesis Kit (Stratagene). All constructs were verified by DNA sequencing. Primers used for generating the mutated *SF3B1* are: (forward) 5′-CTGGTGGATGAGCAGCAGGAGGTCAGAACCATCTCTGC-3′ and (reverse) 5′-GCAGAGATGGTTCTGACCTCCTGCTGCTCATCCACCAG-3′.

### BP mutant constructs

Mutations of potential BP in *TMEM14C* and *ENOSF1* ExonTrap constructs were introduced using QuikChange II Site Directed Mutagenesis Kit (Stratagene) and verified by DNA sequencing. The primer sequences used to generate the mutations are provided in Supplementary Table 3.

### Cell culture and transfection

Mel202 cell line was purchased from the European Searchable Tumour Line Database (Tubingen University, Germany) and MP41 (derived at Institut Curie and described in ref. 31) UM cell lines were cultured in RPMI-1640 supplemented with 10% fetal bovine serum. A point mutation in *SF3B1* resulting in K666T amino-acid substitution was introduced using CRISPR/CAS9-stimulated homology-mediated repair to generate isogenic HEK293T cell lines and was verified by Sanger sequencing. A donor template encoding a puromycin selection cassette was transfected at a 1:1:1 ratio with Cas9 (Addgene 41815) and a *SF3B1*-specific gRNA (built from gRNA cloning vector, Addgene 41824). The selection cassette was removed by flippase-mediated excision. All cell lines were tested and proved to be Mycoplasma free. Authentication of the cell lines was verified by Sanger sequencing for their mutational status and by RNA-Seq.

Plasmid transfections were carried out in cell lines using 500 ng of plasmid construct and LipofectAMINE 2000 reagent (Invitrogen) according to the manufacturer’s instructions. After 24 h, total RNA was extracted with NucleoSpin RNA kit (Macherey-Nagel). The quantity and quality of RNA was determined by spectrophotometry (NanoDrop Technologies). Five hundred nanograms of RNA was used as a template for cDNA synthesis with the High Capacity cDNA Reverse Transcription Kit (Applied Biosystems). Twenty-five nanograms of the synthesized cDNA was used as a template for RT–PCR amplification with specific primers.

HEK293T and MP41 cells were transfected with the following siRNA obtained from Qiagen: *SF3B1* (Cat.No. SI00715932 and Cat. No. SI04154647), *U2AF1* (Cat.No. SI04158049 and Cat. No. SI04159547); *U2AF2* (Cat.No. SI00754026 and Cat. No. SI04194498) or control siRNA (Cat.No. S103650318). The cells were transfected with 50 nM of siRNA using lipofectamine RNAiMAX (Invitrogen). After 48 h, total RNA was extracted and was used as a template for cDNA synthesis. Twenty-five nanograms of the synthesized cDNA was used for RT–PCR amplification with specific primers. PCR products were separated on a 2–3% agarose gel (Supplementary Figs 6 and 7).

### Immunoblot analysis

Cells were lysed in radioimmunoprecipitation assay buffer, and proteins were quantified using a BCA Protein Assay (Pierce). Equal amounts were separated on SDS–polyacrylamide gel electrophoresis gels. Proteins were transferred to nitrocellulose membranes followed by immunoblotting with specific primary antibodies for SF3B1 (1:1,000; #170854; Abcam), Flag (1:1,000, #3165; Sigma), U2AF1 (1:500; #19961; Santa Cruz Biotechnology), U2AF2 (1:500; #53942; Santa Cruz Biotechnology) and β-actin (1:2,000; #5313; Sigma). The membrane was then incubated at room temperature for 1 h with either goat anti-rabbit or goat anti-mouse Odyssey secondary antibodies coupled to a 700 or 800 nm. Immunolabelled proteins were detected using the Odyssey Infrared Imaging System (Li-cor). Quantifications were performed using the ImageJ Software. β-Actin immunoblotting was used to quantify and normalize results.

### Co-immunoprecipitation

HEK293T cells were co-transfected with pcDNA3.11.Myc-U2AF2, kindly given by Edwin Chan^32^, and either pCMV-3tag-1A-SF3B1^WT^ or pCMV-3tag-1A-SF3B1^K700M^. After 48 h, proteins immunoprecipitated by anti-Flag gel affinity (A2220, Sigma) were separated by SDS–polyacrylamide gel electrophoresis and probed in a western blot assay with anti-U2AF2 and anti-U2AF1 antibody (Supplementary Fig. 8).

### Fragment analysis by capillary electrophoresis

Minigene fragments were amplified by RT–PCR using a 5' FAM-forward primer and reverse-specific primers (Supplementary Table 2). One microlitre of RT–PCR product was added to 18.5 μl of deionized formamide and 0.5 μl HD400 marker (Applied Biosystems). The mixture was then denatured 3 min at 95 °C, immediately put on ice, and separated using an ABI 3130xl Genetic Analyzer. The data were analysed using GeneMarker software (SoftGenetics).

## Additional information

**Accession codes**: The RNA-seq data have been deposited in the ArrayExpress repository under the accession code E-MTAB-4097.

**How to cite this article**: Alsafadi, S. *et al*. Cancer-associated *SF3B1* mutations affect alternative splicing by promoting alternative branchpoint usage. *Nat. Commun.* 7:10615 doi: 10.1038/ncomms10615 (2016).

## Supplementary Material

Supplementary Information Supplementary Figures 1-8 and Supplementary Tables 1-3.

Supplementary Data 1 Differential splice junctions of SF3B1-mutant versus SF3B1-wild-type uveal melanoma tumors.

## Figures and Tables

**Table 1:** Sensitive and insensitive 3′ss selected for validation in cell lines.

Gene ID	Position	Alternative splice ratio ([AG'/AG]*100)			
		Wild-type *SF3B1*		Mutated *SF3B1*	
		MP41	HEK293T	Mel202	*SF3B1*^*K666T*^ HEK293T
*Sensitive 3'ss*					
* ENOSF1*	chr18:683,349-683,440	3	3	60	23
* TMEM14C*	chr6:10,724,123-10,725,452	7	8	69	43
* DPH5*	chr1:101,458,237-101,458,383	1	1	66	15
* ZNF76*	chr6:35,257,930-35,258,130	3	2	41	12
* DLST*	chr14:75,356,550-75,356,612	1	1	33	14
* CHTF18*	chr16:844,008-844,060	0	1	55	24
* ARMC9*	chr2:232,209,626-232,209,696	0	0	65	26
					
*Insensitive 3'ss*					
* WRAP73*	chr1:3552508-3551858	0	0	0	0
* VPS45*	chr1:150044293-150048311	0	0	0	0
