# Supplementary material for: Cancer-associated SF3B1 mutations affect alternative splicing by promoting alternative branchpoint usage
Source: Nat Commun. 2016 Feb 4;7:10615. doi: 10.1038/ncomms10615 (PMC4743009; doi:10.1038/ncomms10615)
Supplement: Supplementary Information — Supplementary Figures 1-8 and Supplementary Tables 1-3. [file ncomms10615-s1.pdf]

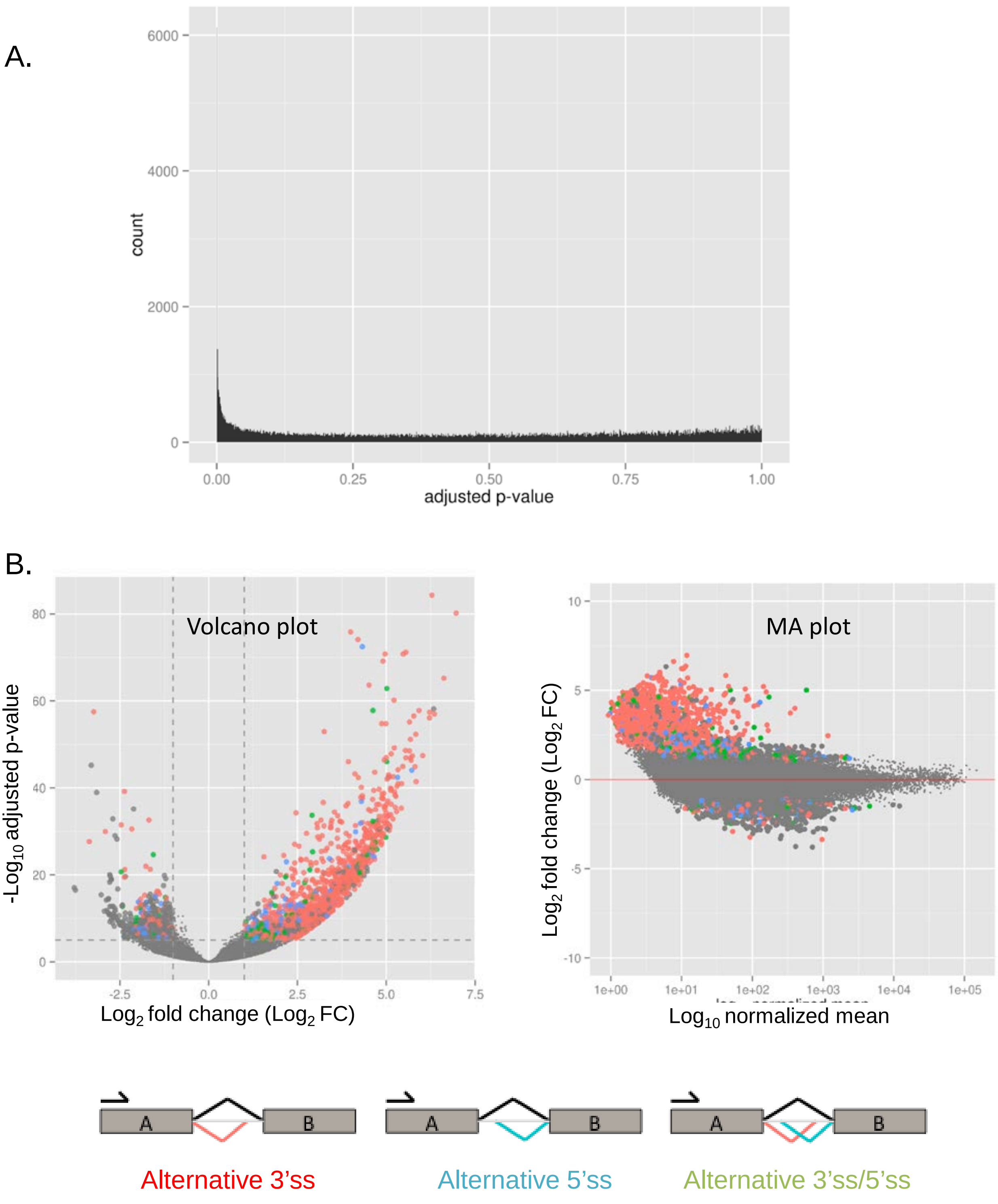

Supplementary Figure 1. RNA-seq differential analysis of splice junctions between the *SF3B1*^{MUT} and *SF3B1*^{WT} uveal melanomas.

A. Adjusted *P* value distribution by gene read in RNA-sequencing dataset**.** The *P* value density plots, stratified by read count quartiles, have a uniform distribution. P values are based on Wald test and Benjamini & Hochberg correction.

B. Volcano plot and MA plot analysis of differential splice junctions. For volcano plot, the logged ratio of splice variant expression between *SF3B1*-wild-type and *SF3B1*-mutant tumors is plotted on the x axis, and false discovery rate (FDR)-adjusted significance is plotted on the y axis (–log10 scale). For MA plot, the logged ratio of splice variant expression is plotted on the y axis, while the the average log intensity is plotted on the x axis. Altered junctions were represented by different colors ; Red: alternative 3’ss; Blue: alternative 5’ss; green: alternative 3’ss/5’ss.

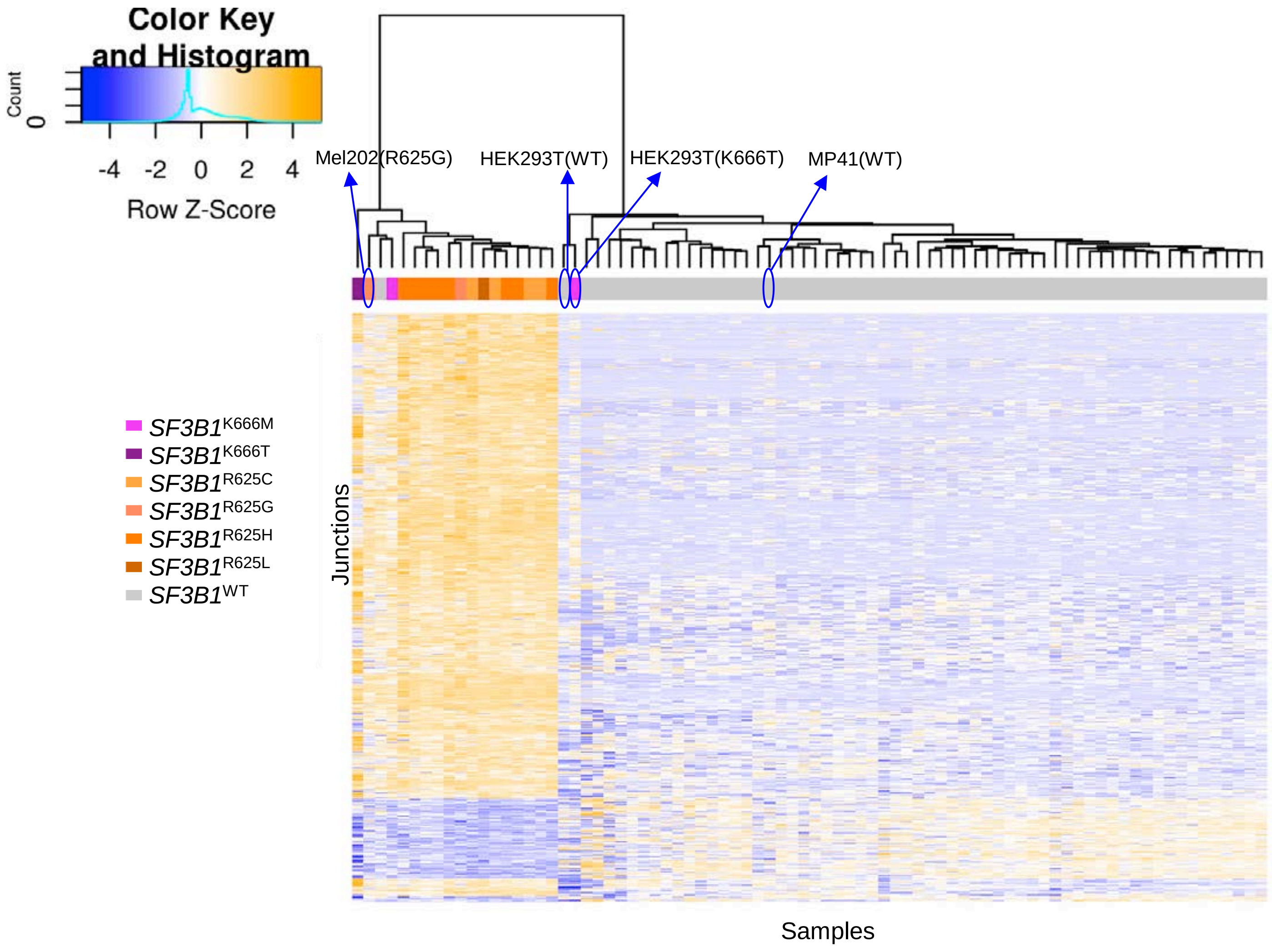

Supplementary Figure 2. Hierarchical clustering and heatmap analysis of differential splice junctions in cell lines and tumor samples.

The colors of squares below the tree denote the subtype of each sample. Below the array tree and the subtype identification row, the heatmap of the 1469 splice junctions is shown.

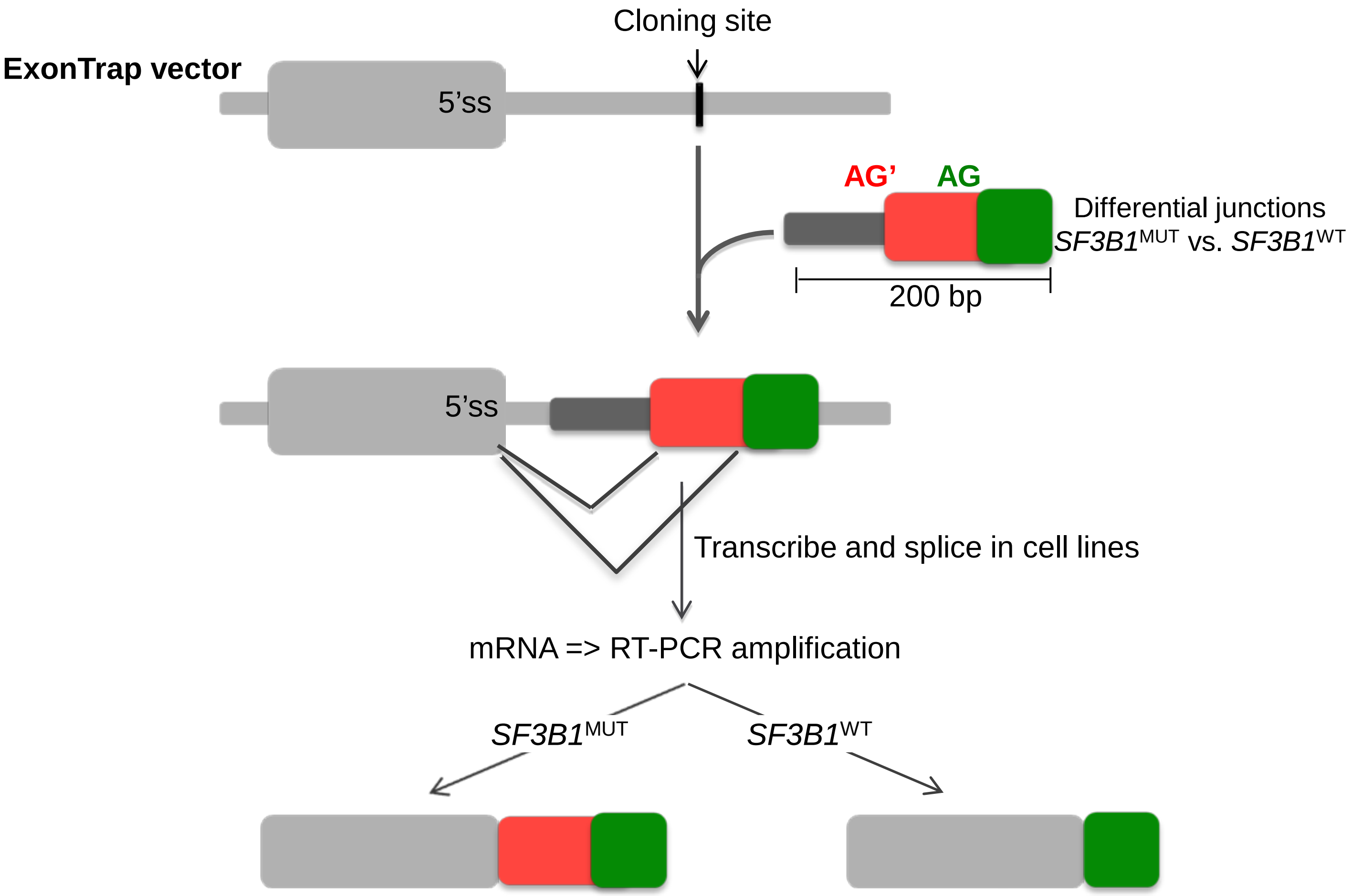

Supplementary Figure 3. Schematic presentation of the minigene splice assay. The ExonTrap vector contains a functional 5' splice site (5'ss) and a multiple cloning site region. AG′-centered sequence of about 200 nucleotides sequences of top differential junctions were cloned in the cloning site. The constructs were then expressed in different cell lines. The different cell lines were transiently transfected with the minigene constructs and examined 48h later for the AG'/AG usage.

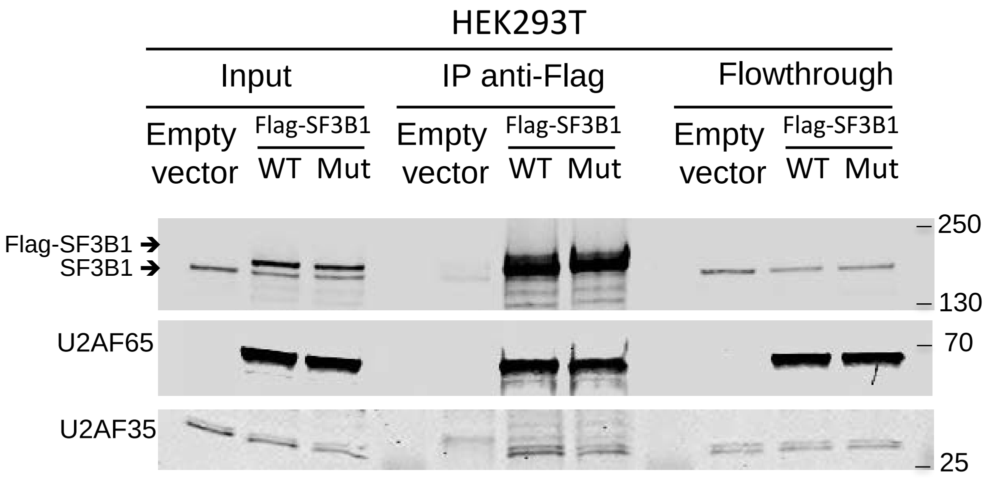

Supplementary Figure 4. Co-immunoprecipitation of U2AF65/35 and wild-type or mutated SF3B1.

HEK293T cells were co-transfected with pcDNA3.11.Myc-U2AF65 and either pCMV-3tag-1A-SF3B1^{WT} or pCMV-3tag-1A-SF3B1^{K700M}. After 48 hours, proteins immunoprecipitated by antiFlag gel affinity were separated by SDS-PAGE and probed in a Western blot assay with anti-Flag, anti-U2AF65 and anti-U2AF35 antibody. Molecular weight markers reported here are visible on over-exposed Supplementary Figure 8.

A.

***TMEM14C* Exontrap cassette**

TGACTGCTGGAGAGCTGTGCTTTTAACTACCTCTGATCCAGCTTGTTTTCTGCAGGTGCAGGCCTG

BP’ BP AG’ AG

***TMEM14C*^{mut1}**

TGACTGCTGGAGAGCTGTGCTTTTAACTAC**tacta**AcCCAGCTTGTTTTCTGCAGGTGCAGGCCTG

***TMEM14C*^{mut2}**

TGACTGCTGGAGAGCTGTGCTTTTAACTAC**tactt**ATCCAGCTTGTTTTCTGCAGGTGCAGGCCTG

***TMEM14C*^{mut3}**

TGACTGCTGGAGAGCTGTGCTT**gct**ACTA**g**CTCTGATCCAGCTTGTTTTCTGCAGGTGCAGGCCTG

***TMEM14C*$^{mut2+3}$**

TGACTGCTGGAGAGCTGTGCTT**gct**ACTA**gtactt**ATCCAGCTTGTTTTCTGCAGGTGCAGGCCTG

***TMEM14C*^{swap}**

TGACTGCTGGAGAGCTGTGCT**tctg**A**t**TACC**ttta**A**c**CCAGCTTGTTTTCTGCAGGTGCAGGCCTG

B.

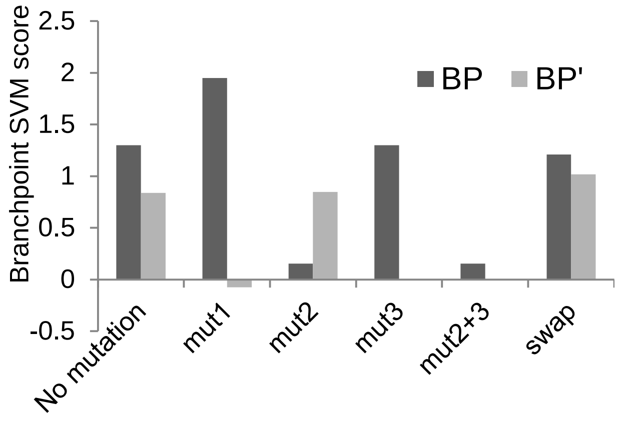

Supplementary Figure 5. Base-pairing potential mutants of branchpoints of *TMEM14C*. A. *TMEM14C* Sequences of site-directed mutagenesis of canonical (BP) and alternative (BP’) branchpoints of *TMEM14C* ExonTrap constructs. B. Predicted SVM scores for the mutated branchpoints.

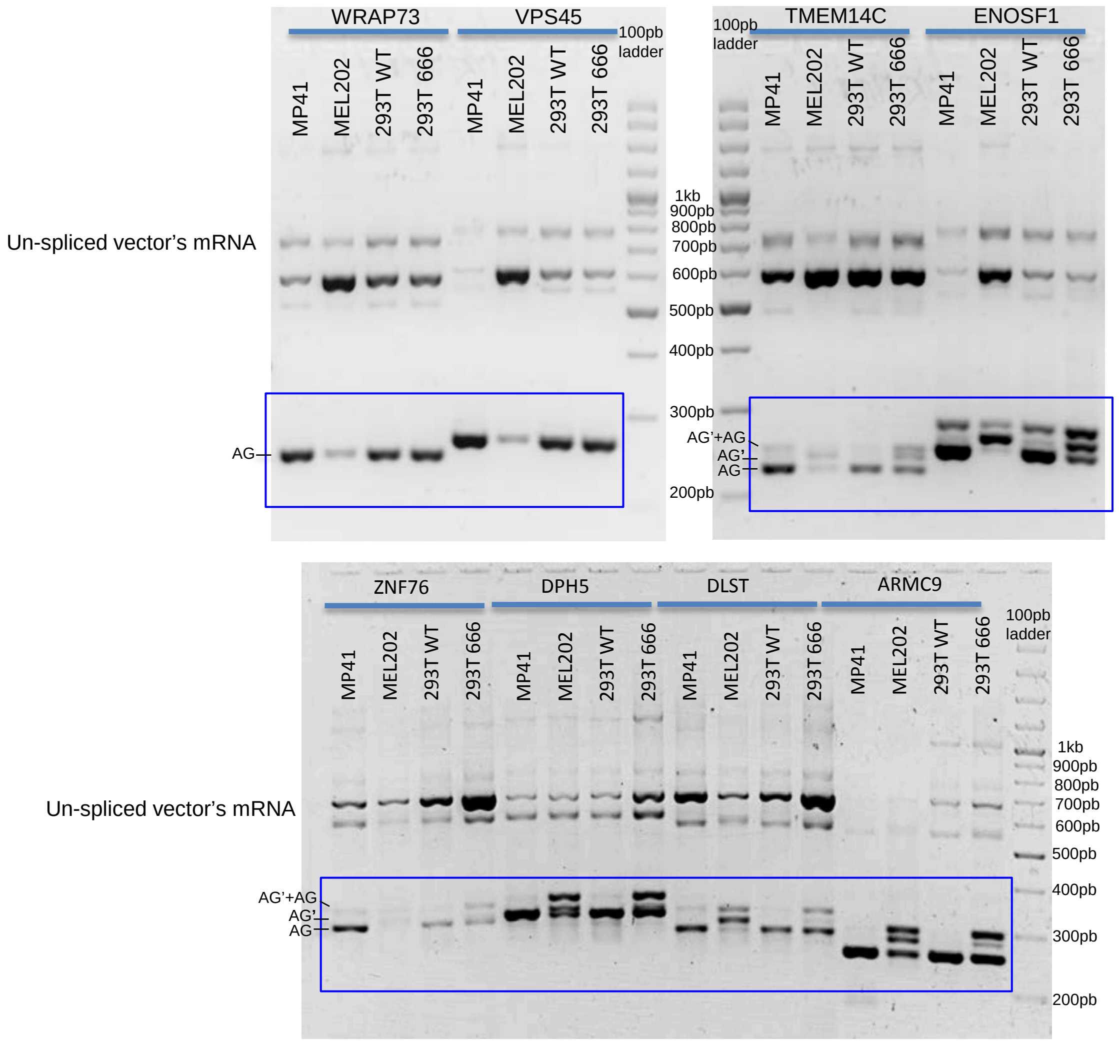

Supplementary Figure 6. Agarose gel full images of Figure 2A

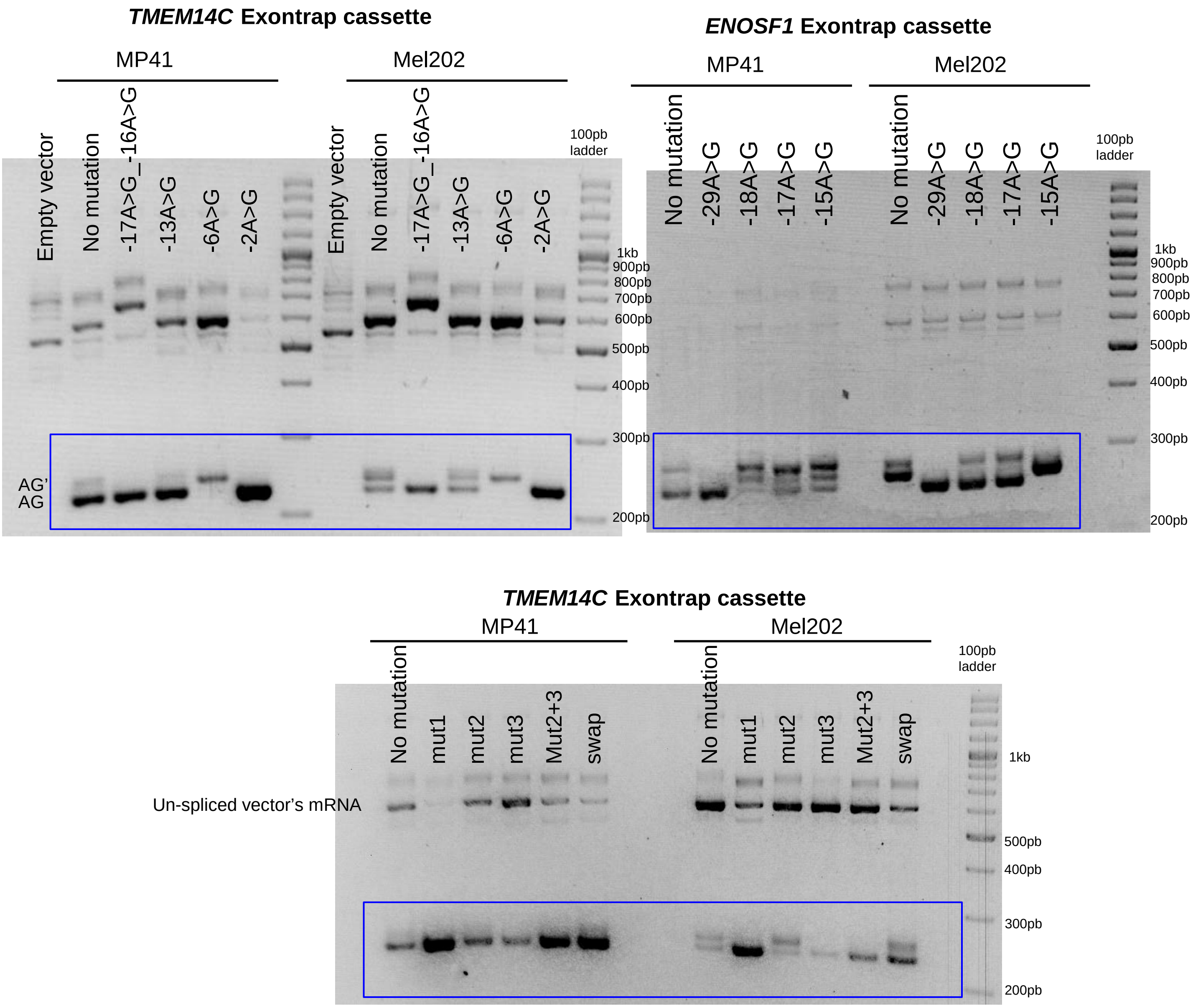

Supplementary Figure 7. Agarose gel full images of Figures 5B and 5C

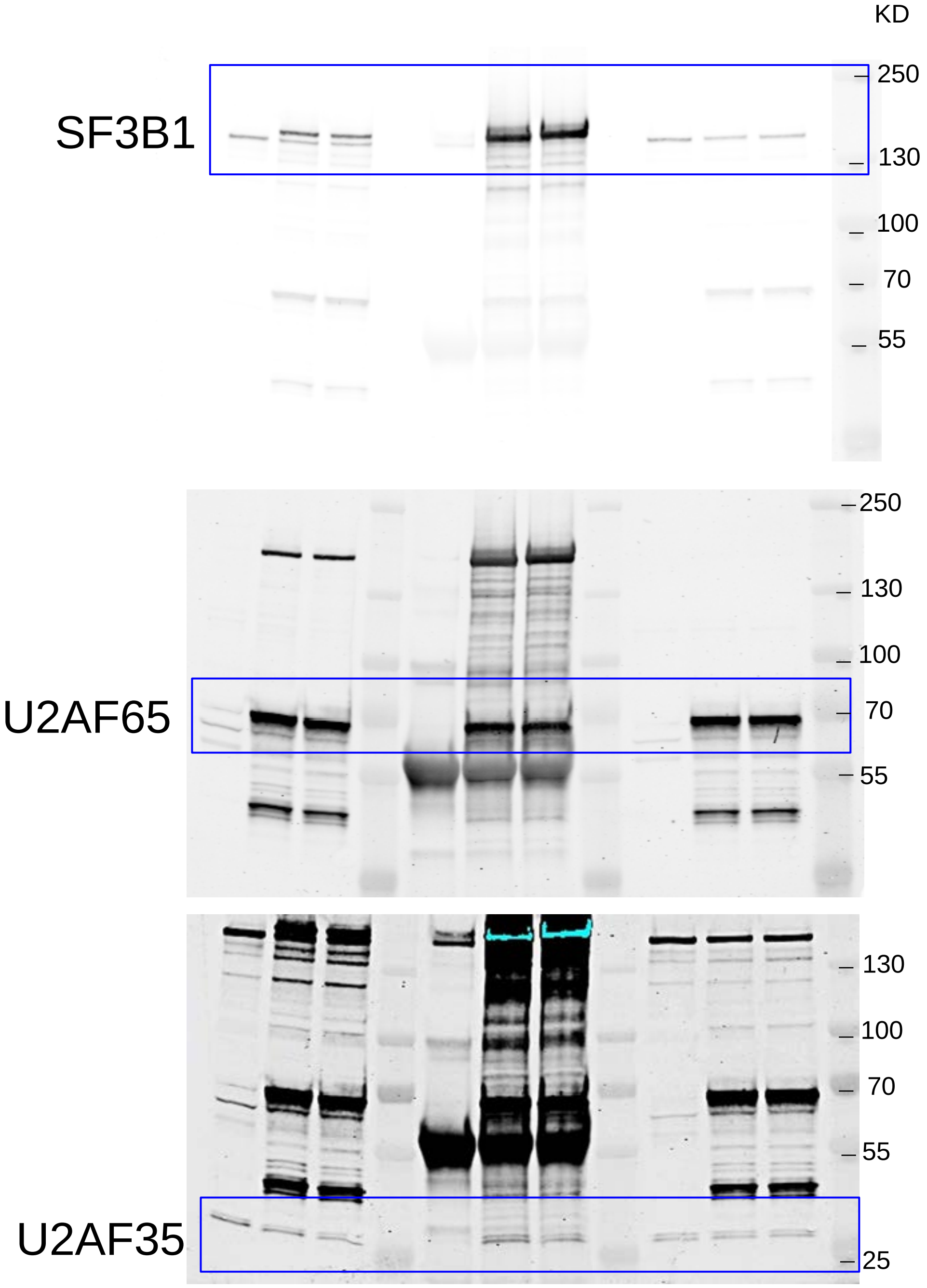

Supplementary Figure 8. immunoblotting row images of Supplementary Figure 4

Supplementary Table 1. Clinical information of 74 patients analyzed by RNA-Seq.

Tumor #	LTD (mm)	Thickness (mm)	TNM	Metastasis	SF3B1 mutation	Histotype	Follow-up (months)	Latest status
1	20	10	T4	No		Spindle cell	95	NED
2	14	13.5	T3	No		Epithelioid cell	73	NED
3	15	12.9	T3	No	p.K666M	Mixed	91	NED
4	20	6.8	T4	Yes		Epithelioid cell	30	DFD
5	11	8.2	T2	No		Epithelioid cell	68	NED
6	10	12.7	T3	No		Epithelioid cell	60	NED
7	13	13.3	T3	No	p.R625C	Spindle cell	68	NED
8	17	16.7	T4	Yes		Mixed	19	DFD
9	24	13.2	T4	No		Spindle cell	69	NED
10	22	14.5	T4	Yes	p.R625H	Epithelioid cell	54	DFD
11	18	14	T4	Yes		Epithelioid cell	19	DFD
12	22	14.8	T4	No	p.R625H	Mixed	31	DFD
13	16	12.1	T4	Yes	p.R625H	Mixed	89	AWD
14	15	11	T3	No	p.R625G	Mixed	61	NED
15	21	13.7	T4	Yes		Epithelioid cell	23	DFD
16	25	12.2	T4	No	p.R625H	Spindle cell	77	NED
17	12	10.2	T3	No		Epithelioid cell	30	DFO
18	18	11.7	T4	No		Spindle cell	81	NED
19	16	13.5	T4	Yes		Epithelioid cell	31	DFD
21	22	10	T4	Yes		Mixed	34	DFD
23	18	11	T4	No	p.R625C	Mixed	57	NED
24	20	15.5	T4	Yes		Spindle cell	61	DFD
25	23	16	T4	No		Mixed	40	NED
26	20	11.5	T4	Yes		Mixed	19	DFD
27	20	11.5	T4	Yes	p.R625C	Spindle cell	30	DFD
28	19.5	12.6	T4	No	p.R625L	Spindle cell	71	NED
29	20	8.8	T4	No		Epithelioid cell	6	DFO
30	21	11.2	T4	Yes		Epithelioid cell	19	DFD
31	18	6.7	T4	No	p.R625H	Mixed	51	NED
32	22	13	T4	Yes		Mixed	9	DFD
33	14	11.3	T3	Yes		Mixed	94	AWD
34	15	14	T3	Yes		Mixed	44	DFD
35	19	12.8	T4	Yes		Epithelioid cell	42	DFD
36	19	12.8	T4	No		Spindle cell	18	DFO
37	21	9.7	T4	No		Epithelioid cell	67	NED
38	16	11.1	T3	No		Epithelioid cell	55	NED
39	18	12.7	T4	No	p.R625H	Epithelioid cell	2	DFO
40	17	12	T3	No		Epithelioid cell	86	NED
41	18	8.3	T4	Yes		Mixed	49	DFD
42	20	8.6	T4	Yes		Mixed	20	DFD
43	14	14.7	T3	Yes		Mixed	50	DFD
44	22	9.1	T4	Yes		Mixed	29	DFD
45	25	13.7	T4	Yes		Mixed	24	DFD
46	20	12	T4	Yes		Mixed	13	DFD
47	23	12.5	T4	Yes		Epithelioid cell	27	DFD
48	19	14.8	T4	Yes		Mixed	39	DFD
49	16	16	T4	No	p.R625H	Mixed	63	DFO
50	18	12	T4	No		Mixed	74	NED
51	22	13.9	T4	No		Mixed	62	NED
52	26	14.8	T4	No		Mixed	13	DFO
53	18	12.6	T4	No	p.R625H	Spindle cell	14	NED
54	12	7.1	T2	No		Spindle cell	66	NED
55	22	12.4	T4	Yes		Spindle cell	17	DFD
56	20	12.5	T4	Yes		Spindle cell	67	DFD
57	19	16	T4	Yes		Mixed	60	DFD
58	15.5	9.8	T3	No		Spindle cell	32	NED
59	22	10.2	T4	Yes		Mixed	30	DFD
60	20	13.1	T4	No	p.K666T	Spindle cell	62	NED
61	16	12.5	T4	Yes		Epithelioid cell	17	DFD
62	15	11.6	T3	Yes		Mixed	29	DFD
63	18.6	12.4	T4	Yes	p.R625C	Mixed	44	DFD
64	18.6	14.8	T4	Yes		Mixed	28	NED
65	20	ND	T4	No		Mixed	61	NED
66	16	8	T3	No		Spindle cell	45	NED
67	16	12.5	T4	No		Spindle cell	74	NED
68	17	13.5	T4	Yes		Spindle cell	85	AWD
69	24	9.1	T4	No		Mixed	63	NED
70	16	12.6	T4	Yes		Mixed	23	DFD
71	21	10.6	T4	No		Epithelioid cell	76	NED
72	15	12.7	T3	Yes		Epithelioid cell	52	DFD
73	20	9.1	T4	Yes		Mixed	79	AWD
74	20	11.2	T4	Yes		Epithelioid cell	25	DFD
75	14.5	11.7	T3	Yes		Mixed	48	DFD
76	13.5	11.6	T3	No		Mixed	45	NED

LTD, Largest tumoral diameter; TNM, stage of disease; AWD, alive with disease; NED, no evidence of disease; DFD, dead from disease; DFO, dead from other cause.

Supplementary Table 2. Primer sequences used for minigene ExonTrap constructs.

minigene	primer sens	sequence 5' 3'
Cntl_WRAP73_F	Forward	GCAGCCCGGGGGATCTCCTGGCGACGGGGGTGT
Cntl_WRAP73_R	Reverse	TAGAACTAGTGGATCCTGTACGTGGACAACAACC
Cntl_VPS45_F	Forward	GCAGCCCGGGGGATCTTGCAGCAGAAATTATCTCC
Cntl_VPS45_R	Reverse	TAGAACTAGTGGATCAGCCACAACTTCCTGTTCA
TMEM14C_F	Forward	GCAGCCCGGGGGATCCAGGTTGGACACACTTCTTTC
TMEM14C_R	Reverse	TAGAACTAGTGGATCTGTCCAGACAGGAGACTACCC
ENOSF1_F	Forward	GCAGCCCGGGGGATCCAGCCCTGCTGTGAGGTT
ENOSF1_R	Reverse	TAGAACTAGTGGATCAGCTTGGACATCCACTCCAC
ZNF76_F	Forward	GCAGCCCGGGGGATCGCCTGTTGGGTCTTAAGCTG
ZNF76_R	Reverse	TAGAACTAGTGGATCTTAAGTGATGAGCGGTGGTG
DPH5_F	Forward	GCAGCCCGGGGGATCCCTGGGAGAGCTAATTGCAT
DPH5_R	Reverse	TAGAACTAGTGGATCTGAGAGCCCTTCTAGTGTCTTG
DLST_F	Forward	GCAGCCCGGGGGATCGCCCTGTAGGAAAGGGTTCT
DLST_R	Reverse	TAGAACTAGTGGATCAAGCCCATGAAAAGCTGGTA
CHTF18_F	Forward	GCAGCCCGGGGGATCGATGGGGCCTCTGAGTGTC
CHTF18_R	Reverse	TAGAACTAGTGGATCGGACACGGTAGAATCGCTGT
ARMC9_F	Forward	GCAGCCCGGGGGATCTGACATGATCCTTCCTCATTT
ARMC9_R	Reverse	TAGAACTAGTGGATCCCGCTCCACTGCACATTAG
FAMExTrap_F	Forward	FAM-GAGGGATCCGCTTCCTGCCCC

Supplementary Table 3. The primer sequences used to generate mutations.

Gene	Primer	Sense	sequence 5' 3'
ENOSF1	-29 (c.805-29A>G)	Forward	tgaggttggctcagggctggcctccctgtgaagagtctc
	-29 (c.805-29A>G)	Reverse	gagactcttcacagggaggccagccctgagccaacctca
	-16 (c.805-16A>G)	Forward	ggctgacctccctgtggagagtctctttttgca
	-16 (c.805-16A>G)	Reverse	tgcaaaaagagactctccacagggaggtcagcc
	-17 (c.805-17A>G)	Forward	gctgacctccctgtgaggagtctctttttgcag
	-17 (c.805-17A>G)	Reverse	ctgcaaaaagagactcctcacagggaggtcagc
	-15 (c.805-15A>G)	Forward	tgacctccctgtgaagggtctctttttgcagatg
	-15 (c.805-15A>G)	Reverse	catctgcaaaaagagacccttcacagggaggtca
TMEM14C	-17_16 (c.-58-17A>G_-58-16A>G)	Forward	ctgctggagagctgtgcttttggctacctctgatccag
	-17_16 (c.-58-17A>G_-58-16A>G)	Reverse	ctggatcagaggtagccaaaagcacagctctccagcag
	-13 (c.-58-13A>G)	Forward	gctggagagctgtgcttttaactgcctctgatccag
	-13 (c.-58-13A>G)	Reverse	ctggatcagaggcagttaaaagcacagctctccagc
	-6 (c.-58-6A>G)	Forward	gtgcttttaactacctctggtccagcttgttttctgcag
	-6 (c.-58-6A>G)	Reverse	ctgcagaaaacaagctggaccagaggtagttaaaagcac
	-2 (c.-58-2A>G)	Forward	tttaactacctctgatccggcttgttttctgcaggtg
	-2 (c.-58-2A>G)	Reverse	cacctgcagaaaacaagccggatcagaggtagttaaa
	mut1	Forward	ggagagctgtgcttttaactactactaacccagcttgttttctgcaggtg
	mut1	Reverse	cacctgcagaaaacaagctgggttagtagtagttaaaagcacagctctcc
	mut2	Forward	tggagagctgtgcttttaactactacttatccagcttgttttctgcag
	mut2	Reverse	ctgcagaaaacaagctggataagtagtagttaaaagcacagctctcca
	mut3	Forward	ctgactgctggagagctgtgcttgctactagctctgatccagcttgttttctg
	mut3	Reverse	cagaaaacaagctggatcagagctagtagcaagcacagctctccagcagtcag
	mut2+3	Forward	tctttctgactgctggagagctgtgcttgctactagtacttatccagcttgttttctgcaggtgcag
	mut2+3	Reverse	ctgcacctgcagaaaacaagctggataagtactagtagcaagcacagctctccagcagtcagaaaga
	swap	Forward	ctttctgactgctggagagctgtgcttctgattacctttaacccagcttgttttctgcaggtgc
	swap	Reverse	gcacctgcagaaaacaagctgggttaaaggtaatcagaagcacagctctccagcagtcagaaag
